# Successful management of acute myeloid leukemia transformed from chronic myelomonocytic leukemia in the elderly by a combination regimen of decitabine and cytarabine, aclarubicin and granulocyte colony-stimulating factor: A case report

**DOI:** 10.3892/ol.2015.2870

**Published:** 2015-01-13

**Authors:** QI DENG, JING-YI LI, PENG-JIANG LIU, MING-FENG ZHAO

**Affiliations:** Department of Hematology, The First Central Hospital of Tianjin, Tianjin 300192, P.R. China

**Keywords:** acute myeloid leukemia, chronic myeloid leukemia, decitabine, cytarabine, aclarubicin and granulocyte colony-stimulating factor, single nucleotide polymorphism

## Abstract

Despite advances in the treatment of acute myeloid leukemia (AML) in recent years, the outcome of elderly AML patients with antecedent hematological disorders remains unsatisfactory. The present study describes a case of complete remission in an elderly patient with AML transformed from chronic myelomonocytic leukemia (CMML) and the treatment of the case with decitabine in combination with cytarabine, aclarubicin and granulocyte colony-stimulating factor (CAG). A 70-year-old male was admitted with fever, pruritus and weakness that had been apparent for two weeks, and a two-year history of monocytosis (22.5–27.0%). Further examinations revealed a hemoglobin level of 106 g/l, a white blood cell count of 39.52×10^9^/l, a platelet count of 81×10^9^/l, Y chromosome loss and uniparental disomy on chromosomes 4q, 2q and 19p. The patient was diagnosed with AML transformed from CMML, with cytogenetic anomalies. A combination regimen of decitabine and CAG was administered. Subsequent to one cycle, the patient achieved complete remission. The patient was then followed up with three courses of the same regimen and achieved clinical remission, with no evidence of AML relapse. The present study suggests that a combination of low-dose decitabine and CAG may offer a novel and potentially effective treatment regimen for elderly AML patients.

## Introduction

Acute myeloid leukemia (AML) refers to a highly heterogeneous group of hematopoietic diseases, which are characterized by clonal accumulation and the growth of immature myeloid cells within the bone marrow. At present, the primary treatment for AML is remission induction therapy with multiple, combined chemotherapeutic agents, which aims to reduce the total body leukemic cell population to an undetectable level. This is usually followed by either consolidation chemotherapy or allogeneic hematopoietic stem cell transplantation (HSCT), depending on how well the patient responds to chemotherapy alone and their response to intensive treatments (1). The benefits of this treatment approach vary considerably with patient age and AML subtype, which are two of the most important prognostic factors. Elderly AML patients, particularly those >65 years old and with a history of myelodysplastic syndromes (MDS) or antecedent hematological disorders, exhibit unsatisfactory tolerance profiles and disappointing complete remission (CR) and overall survival rates following this conventional approach (2–4). As AML predominantly occurs in older individuals and the global population is rapidly aging, novel treatment strategies are urgently required for elderly patients with AML (5). The present study describes a case of CR without relapse nine months after the administration of a combination regimen of decitabine and cytarabine, aclarubicin and granulocyte colony-stimulating factor (G-CSF) (CAG) in an elderly patient with AML transformed from chronic myelomonocytic leukemia (CMML) (6).

## Case report

**Figure 1:**
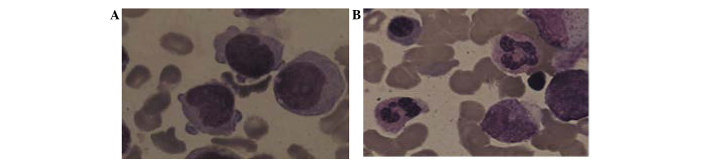
Digital images revealing Wright and Giemsa-stained bone marrow monoblasts and promonocytes (A) prior to and (B) following treatment with the combination regimen (magnification, ×1,000).

**Figure 2:**
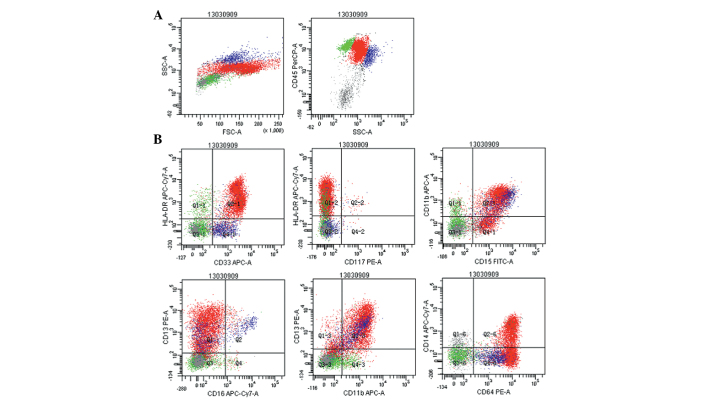
Flow cytometry scatter plots revealing the gating strategy with (A) cluster of differentiation (CD)45/side-scattered light and (B) the ratio of monoblasts (72.3%), and their expression of the myelomonocytic antigens, human leukocyte antigen-DR, CD15, CD4, CD13, CD33, CD38, CD64, D11b, CD14 and CD56.

**Figure 3:**
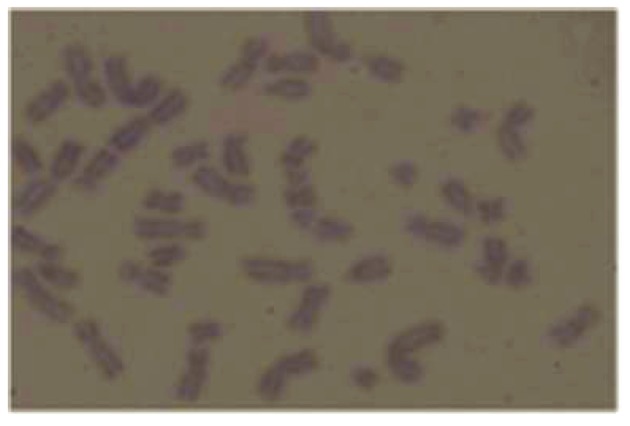
Cytogenetic analysis revealing Y chromosomal loss, which was the single anomaly identified in the patient (45,X,−Y[4]/46,XY[16]) (magnification, ×1,000).

**Figure 4:**
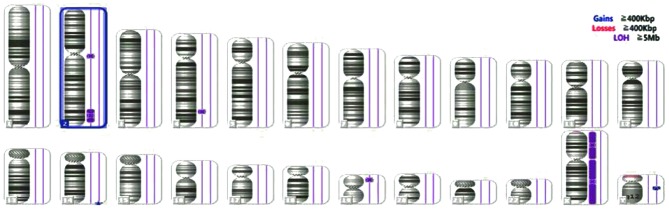
Uniparental disomy of chromosomes 4q, 2q and 19p, and a copy number variation of 14q. Yq chromosome deletion detected by single nucleotide polymorphism analysis. LOH, loss of heterozygosity.

In March 2013, a 70-year-old male presented to The First Central Hospital of Tianjin (Tianjin, China) with fever, pruritus and weakness that had been apparent for two weeks, and a two-year history of monocytosis (22.5–27.0%). The inpatient laboratory tests revealed a hemoglobin (Hb) level of 120 g/l, a white blood cell (WBC) count of 9.23×10^9^/l and a platelet count of 122×10^9^/l, but an abnormal monocyte percentage of up to 35.5% (normal range, 5–10%). The anti-inflammatory drug, piperacillin-tazobactam (4.5 g, once every 12 h), was administered for three days, however, no improvement was noted with regard to the symptoms of pruritus and weakness. The patient was therefore admitted to The First Central Hospital of Tianjin. Upon admission, a physical examination revealed rashes on the extremities, but no sternal pain or tenderness, and no peripheral lymphadenopathy or hepatosplenomegaly. The routine blood test identified a Hb level of 106 g/l, a WBC count of 39.52×10^9^/l and a platelet count of 81×10^9^/l. A diagnosis of AML was suspected. A bone marrow aspirate examination revealed a monoblast percentage of 38% and a promonocyte percentage of 55% (Fig. 1A), values which were significantly higher than the threshold of blasts (>20%) required in the bone marrow for a diagnosis of AML, according to the 2008 World Health Organization (WHO) (6) AML classification systems. Immunohistochemical analysis revealed strong staining signals for myeloperoxidase in the cytoplasm of the blasts, and the myeloid lineage markers, human leukocyte antigen-DR and cluster of differentiation (CD)15, CD4, CD13, CD33, CD38, CD64, CD11b, CD14 and CD56, were detected on the surface of the monoblasts by multiparameter flow cytometry (Fig. 2). There was no evidence of a JAK2V617F mutation or a BCR/ABL fusion transcript. In addition to the bone marrow aspiration assessment, immunohistochemical staining and flow cytometric analyses, single nucleotide polymorphism (SNP) arrays were performed. Cytogenetic abnormalities, including Y chromosome loss (Fig. 3) and uniparental disomy (UPD) on chromosomes 4q, 2q and 19p (Fig. 4) were detected. Considering the two-year history of monocytosis, the patient was diagnosed with AML transformed from CMML together with a rare genetic abnormality (6).

The patient was treated with 1,000 mg imipenem every 8 h for eight days, and 500 mg hydroxycarbamide every 12 h for four days. Subsequently, a combination treatment regimen consisting of decitabine and CAG was initiated. In total, 15 mg/m^2^ decitabine was administered daily on days one to five, 20 mg aclarubicin daily on days three to six, 20 mg/m^2^ cytarabine twice daily on days three to 16 and 150 μg G-CSF daily from day two until the attainment of a normal WBC count. At the end of the low-dose regimen, the patient achieved a CR and experienced less severe adverse events, specifically severe infections. The values of the major test parameters were normal or close to normal, and included a Hb level of 117 g/l, a WBC count of 4.52×10^9^/l, a platelet count of 370×10^9^/l, a monocyte percentage of 6.5% and a monoblast percentage of 1.5% (Fig. 1B). Administration of the regimen was continued. Subsequent to four cycles of treatment with decitabine in combination with CAG, the patient achieved a successful clinical remission and demonstrated no evidence of AML relapse for nine months. Written informed consent was obtained from the patient’s family for the publication of the case report and the accompanying images.

## Discussion

CMML, defined by the 2008 WHO classification of myeloid neoplasms as a clonal hematopoietic stem cell disorder (6), is currently classified under a new division of myeloid neoplasms, the MDS/myeloproliferative neoplasm disorders. These disorders exhibit dysplastic and proliferative features. Cytogenetics is considered to be one of the most valuable determinants used during the risk classification and prognostication of AML (7,8). Previous studies have revealed that SNP arrays can identify chromosomal markers that cannot be detected by conventional cytogenetics (9,10). In general, specific cytogenetic alterations cannot be identified in patients with CMML by chromosome banding analysis alone. The most common single chromosomal abnormalities in cases of CMML are monosomy 7 (3.9–8.5%), trisomy 8 (4.1–7.8%) and other abnormalities, which may include complex karyotypes (4.4–6.3%), isochromosome 17 (1–2%), trisomy 21 (1–2%) and deletion of 5q (1.5%) (11–13). Abnormal karyotypes have been reported in 20–40% of CMML cases, and include abnormalities shared with other myeloid neoplasms (14). According to a previous study involving 140 patients (10), acquired somatic UPD is not uncommon in primary (29%) and secondary (35%) AML. Although AML with UPD q4 is rarely reported (15), another previous study (16) revealed an association between UPD 4q and CMML. Based on the aforementioned criteria and studies, the patient in the present study was diagnosed with AML transformed from CMML.

CMML remains to be a challenging malignancy to treat, and for this reason, the median survival rate for patients ranges between 12 and 18 months. If CMML progresses to AML, patients exhibit poor prognoses, face limited treatment options and on average, demonstrate survival rates of only a few months (17). The only potential curative treatment for patients with CMML is allogeneic HSCT, however, this particular approach is usually unsuitable for elderly individuals. Treatment with the DNA methyltransferase inhibitors, azacitidine and decitabine, has been extensively studied for the management of AML and MDS (18–21). Two mechanisms are believed to underlie the action of decitabine. First, decitabine incorporates into the DNA following phosphorylation, without the need for reduction. Secondly, decitabine does not incorporate into RNA, and inhibits DNA methyltransferase 2 (22). A Japanese study, which included AML patients ≥60 years old, revealed that when a 10-day decitabine regimen was administered and then repeated following an interval of 4–5 days, CR and median survival rates of 47% and 12.7 months, respectively, were achieved subsequent to an average of three cycles of therapy (23). The CAG regimen, which combines G-CSF with low doses of cytarabine and aclarubicin, was first used by a Japanese study in 1995, and was reported to be a chemotherapy option for AML (24). In this regimen, aclarubicin is effective regardless of multi-drug resistance gene status, and G-CSF acts to enhance the transition of resting G_0_ phase AML cells into the cell cycle. The CAG regimen has been used for relapsed or refractory AML and MDS patients, and also specifically for elderly patients. The high response rates and good tolerability of this regimen is observed not only in relapsed or refractory cases, but also in previously untreated patients (25,26). Therefore, for these reasons, the combination of decitabine and CAG was selected to treat the patient in the present study.

Due to the conjecture diagnosis of CMML in the present study, the regimen of decitabine in combination with CAG was selected. This regimen consisted of low-dose decitabine (15 mg/m^2^), aclarubicin (15 mg/m^2^ for four days) and low-dose cytarabine (15 mg/m^2^, every 12 h for 10 days). The patient achieved a CR following only one such low-dose regimen, and experienced less severe adverse events, specifically severe infections. Subsequent to a total of four cycles of treatment with decitabine in combination with CAG, the patient achieved a successful clinical remission. In conclusion, a combination of low-dose decitabine and CAG may offer a novel and potentially effective treatment regimen for cases of AML transformed from CMML.
